# Collaborative Strategies to Improve Nutrition Security and Education: Lessons Learned During a Pandemic

**DOI:** 10.1111/josh.13247

**Published:** 2022-10-19

**Authors:** Ashley Rosales, Shannan Young, Tracy Mendez, Kristal Shelden, Megan Holdaway

**Affiliations:** ^1^ Dairy Council of California Sacramento CA; ^2^ Dairy Council of California Sacramento CA; ^3^ Dairy Council of California Sacramento CA; ^4^ Dairy Council of California Sacramento CA; ^5^ Dairy Council of California Sacramento CA

**Keywords:** nutrition education, school meals, food access, COVID‐19 pandemic, nutrition security, collaboration, community nutrition

## Abstract

**BACKGROUND:**

The COVID‐19 pandemic intensified disparities for underserved populations as accessing resources became more difficult. Dairy Council of California launched the Let's Eat Healthy initiative to address nutrition security through collaborative solutions in the school environment.

**IMPLICATIONS FOR SCHOOL HEALTH POLICY, PRACTICE, AND EQUITY:**

To ensure nutrition security for children and families, nutritious food and nutrition education must go hand‐in‐hand. Improving access to high quality food can help address the health disparities that exist for people who are at increased risk for food insecurity. Nutrition education supports students' holistic learning and social and emotional learning skills. Nutrition education models must be increasingly flexible in the face of ongoing challenges. Collaborative efforts to connect food access hubs, such as schools, with support and resources to provide evidence‐based nutrition education and agricultural literacy can equip individuals and communities with the knowledge, skills, and ability to make nutrient‐rich food choices.

**CONCLUSIONS:**

Investments and strategies in nutrition security that utilize the Individual plus Policy, System, and Environmental (I + PSE) model, such as the Let's Eat Healthy initiative, will effectively influence positive behavior change and improve community health. Navigating challenges in a rapidly changing environment requires people and organizations to work together, across disciplines, to leverage knowledge, experience, resources, expertise, and creative thinking. Improving access to healthy food and nutrition education will be most effective when done through collaboration.

## BACKGROUND

The onset of the COVID‐19 pandemic in 2020 upended daily life across the world, impacting people's physical, social, and emotional health. The ongoing public health crisis intensified disparities for underserved populations as access to resources such as food, education, healthcare, and technology became more difficult. Providing vulnerable populations, particularly children, with nutrition security and education became critical needs, as childhood nutrition affects the ability to succeed in school and life and can also determine future health outcomes, including mental and emotional well‐being and risk of developing chronic diseases.^1^, ^2^, ^3^ Schools are the heart of communities, providing services and a critical safety net offering families support, resources, and healthy environments where children are nurtured to grow optimally, learn, and thrive.

Dairy Council of California is a nutrition education organization that collaborates with other local, state, national, and international organizations and agencies to elevate the health of children and families through the pursuit of lifelong healthy eating habits. The organization's science‐based nutrition education resources, Farm to School assemblies, professional development programs, and online resources educate millions of children and families in California and throughout the United States.

More than 2 years after the onset of the COVID‐19 pandemic, there is opportunity to reflect on the challenges it raised and learn from the solutions it inspired. Collaboration can accelerate new pathways to improve the health of children, families, and communities. Effective collaboration can support optimizing school meal programs, provide nutrition education, and increase access to nutritious foods. When organizations collaborate, they have the capacity to accomplish far more than any 1 organization could do on its own. This article documents the effect of strategically partnering with education, health, agriculture, and other sectors to improve nutrition education and healthy food access in the school environment.

## LET'S EAT HEALTHY INITIATIVE

In June 2020, Dairy Council of California launched Let's Eat Healthy, an initiative that brings together educators, health professionals, and community leaders to elevate the health of children and families through the pursuit of lifelong healthy eating habits. Let's Eat Healthy invites multidisciplinary coordination, collaboration, and co‐creation to champion community health by teaching and inspiring healthy eating habits and making healthy, wholesome foods accessible and affordable to all California's diverse communities.

The Let's Eat Healthy initiative is grounded in the Individual plus Policy, System, and Environmental (I + PSE) model.^4^ I + PSE addresses adaptive challenges and supports multidimensional strategies and evaluation using a collaborative approach to implement policies, systems, and environments that promote nutrition and health while strengthening individual knowledge and behavior. Nutrition education is part of a larger systems approach to health, delivered through schools, communities, and policies. It exists in a variety of learning settings, from classrooms to gardens to community centers. Nutrition education, when supported by policy, system, and environmental interventions, serves as a strong catalyst to elevate the health of communities.

## SCHOOL MEAL ACCESS

Schools support children's overall health and well‐being by providing nourishing meals, especially for those living in socioeconomically disadvantaged communities or food‐insecure homes. Student participation in school meal programs is associated with higher intake of fruits, vegetables, whole grains, and dairy foods.^5^ These food groups support the intake of important nutrients such as calcium and fiber that are typically under consumed in the American diet,^2^ making school meals a vital source of nutrition for many children. Recent research from Tufts University found that schools were the healthiest source of food consumed across a sample of children and adults in the United States.^6^ The study looked at patterns and trends in diet quality by food source—including schools, restaurants, and grocery stores—and found that diet quality for foods served at schools improved significantly from a similar study conducted 14 years earlier.

The abrupt closure of schools at the onset of the COVID‐19 pandemic disrupted meal service for millions of children, creating substantial challenges for parents and caregivers of children that rely on school meals. To support families and school communities, Dairy Council of California partnered with the California Milk Processor Board to develop a landing page for information on meal sites and a statewide public awareness campaign to reach families with this important information. The site provided information on school meal sites across the state and eventually expanded to include food bank locations and distance learning nutrition resources.

The campaign and landing page were shared via social and traditional media channels with the support of California State Senator Dr. Richard Pan, No Kid Hungry—a child hunger nonprofit organization—and the California Department of Education, as well as well‐known actors and sports figures. Together, the campaign and landing page became a valued resource for California school districts and communities, supporting 528,797 website visits, including 467,823 unique views. Approximately 40% of viewers sought information in Spanish. This effort to ensure students with the greatest need received healthy meals was effective because of the collaborative, innovative efforts of individuals and organizations. In part due to efforts such as this, participation in the National School Lunch Program in 2020 declined less in the state of California than the national average.^7^

## NUTRITION EDUCATION INNOVATION

In addition to reliable sources of nutritious foods, all children and families deserve access to nutrition education, which includes basic knowledge and skills to successfully navigate a complex world of food choices and food topics. Teachers can make an important contribution to the knowledge and dietary habits of children,^8^ and these learned skills and positive health behaviors support physical, social, and emotional health, as well as academic success.^9^ Despite the positive role nutrition and nutrition education play in overall health, the pandemic made nutrition education challenging for teachers and public health educators. For example, the Let's Eat Healthy K‐12 curriculum is traditionally taught in a classroom setting, which was not a possibility in many classrooms throughout California for most of the 2020‐2021 school year.

Through partnerships with educators, Dairy Council of California adapted its Let's Eat Healthy K‐12 resources to ensure that children and families across California were supported with nutrition education during the pandemic. Shifting to remote education models enabled teachers to continue instructing children and families on healthy eating patterns that include nutritious, high‐quality foods. Adaptations included a variety of co‐created online resources that easily embed into online learning platforms featuring digital documents, short and informative videos, and grade‐appropriate quizzes and games. A new Technology Tutorial Guide walked educators through the process of downloading and embedding resources into online learning platforms like Google Classroom and Zoom or apps like SeeSaw for quick and easy implementation. The collaborative effort with educators to adapt resources for online learning and continue teaching nutrition despite the challenge of not meeting in person enabled 4.4 million California students and families to engage with Let's Eat Healthy nutrition resources during the 2020‐2021 school year. Moving forward, virtual resources remain accessible to children in both traditional and nontraditional learning environments, as well as those outside of the school environment.

The shift to remote learning also applied to Dairy Council of California's Farm to School program, Mobile Dairy Classroom (MDC). MDC is an assembly that brings the farm experience to students, teaching them how milk and dairy foods get from the farm to the table and how they contribute to a healthy eating pattern. During the 2020‐2021 school year, MDC collaborated with the California Milk Advisory Board to begin innovative virtual field trips, combining the traditional experience of an assembly with a virtual farm tour. Over 225,000 students, families, and classrooms engaged with dairy farmers and agriculture instructors through a livestream viewing from the farm. Beyond the pandemic, virtual field trips will continue to be an important tool to increase access to educational opportunities for students who may not otherwise be able to participate. Teaching nutrition through Farm to School programs helps bridge the knowledge gap between agriculture and the food students eat, resulting in a greater awareness of the role agriculture plays in a healthy diet, while a virtual format makes experiential learning accessible to more students, families, and communities around the world.

The pandemic increased the need for resources that address the whole child, paving the way for nutrition education to support students beyond developmental and physical health to also include social and emotional health. Let's Eat Healthy nutrition resources include embedded social and emotional learning (SEL) strategies, making them relevant to school priorities. SEL competencies that can be addressed through nutrition education include increasing social awareness through agriculture literacy, building self‐awareness around food choices, practicing self‐management and responsible decision making through nutrition knowledge, and improving relationship skills by respecting cultural differences in eating habits. Nutrition education materials that include SEL strategies support healthier students, schools, and communities.

## INTEGRATING FOOD ACCESS AND NUTRITION EDUCATION

Evidence‐based nutrition education provides individual knowledge and skills, and it can be leveraged to address food security outside the school environment. One example is the efforts led by Scott Brown, a P.E. teacher from an area where a high proportion of students experience food insecurity. With interactive technology and the Let's Eat Healthy nutrition curriculum, Brown incorporated questions into online lessons that assessed his students' ability to access healthy foods. Through Brown's trusted relationship with students and a drive to support healthy eating habits, he successfully bridged education and food access. As a result, the school partnered with a local church and the district's food services to provide free groceries for 13 students' families. This was in addition to 42 students who started participating in the school meal program.

Another example of integrating nutrition education and healthy food access into existing systems is the Summer STARS program, a partnership between Dairy Council of California, Community Housing Opportunities Corporation, and United Way California Capital Region (United Way). The goal of this partnership was to reduce learning and achievement gaps in children and to ensure students were mentally and physically prepared to resume school after summer break. Through partnerships, 175 students engaged in Let's Eat Healthy nutrition lessons, strengthening reading skills while gaining nutrition literacy. In addition, local school districts participated in summer meal programs along with the enrichment activities at some sites to ensure students had access to the nutritious foods they were learning about. As a result of Summer STARS intervention, United Way reported gains in overall literacy and reading levels of participating students. Through enrichment activities in Let's Eat Healthy nutrition education resources, students reinforced reading and writing while also learning nutrition literacy and building essential life skills.

## CONVENING TO IMPROVE CHILDHOOD NUTRITION

Partnerships built on shared values are essential to improve nutrition security and thus the overall health of children and families. Well‐Nourished, Brighter Futures, a statewide convening in January 2021 to grow the Let's Eat Healthy initiative in California, aims to improve nutrition education, facilitate and provide access to nutritious foods such as fruits, vegetables, whole grains and milk and dairy foods, and advocate for all children's nutritional needs. Together with The Children's Partnership, Latino Coalition for a Healthy California, First 5 Sacramento, Los Angeles County Office of Education, and No Kid Hungry brought together passionate experts in health representing community, academic, government, and advocacy organizations in California. Participants contributed to the development of the initiative's strategic framework report, including objectives and action steps. Additionally, the convening engaged participants in cross‐sharing, learning, and the identification of next steps for the initiative to address issues of nutrition equity in California communities.

The convening highlighted the importance of ensuring children's health is supported right from the start. As a result, addressing the gaps in support for optimal nutrition during pregnancy and infancy through age 2 years, known as the first 1000 days of life, can go a long way in helping children grow healthfully, making them better prepared to learn and thrive. A primary recommendation of the Well‐Nourished, Brighter Futures convening was to assess current resources, services, and unmet needs during the first 1000 days of life. To work toward this objective, a subgroup received grant funding from University of California, Irvine to conduct a community needs assessment of nutrition support and resources throughout the first 1000 days of life in low‐income families in California. This academic‐community partnership continues the work of the convening's strategic objectives for a comprehensive and coordinated approach to improve nutrition security and education to support health outcomes in early life development.

## IMPLICATIONS FOR SCHOOL HEALTH POLICY, PRACTICE, AND EQUITY

To ensure nutrition security for children and families, nutritious food and nutrition education must go hand‐in‐hand. Schools need additional resources to support the physical, social, and emotional health of their students as they continue to navigate the challenges brought on by the COVID‐19 pandemic. Improving access to high quality food can help address the health disparities that exist for people who are at increased risk for food insecurity. Food insecurity and poor diet quality are closely linked, and food insecurity is associated with numerous negative health consequences. Childhood nutrition affects physical growth and future disease risk, as well as academic success, attendance, and behavior in school. Nutrition education equips students with the knowledge, skills and ability to make healthier food choices while also supporting holistic learning and SEL skills, teaching them to make connections with food, health, environment, and community.

Ongoing challenges in the school environment create a need for nutrition education models to be increasingly flexible and include more multidimensional strategies that extend beyond the classroom. Cross‐sector collaboration is key to bridging the gap between nutrition education and the adoption of healthy dietary patterns. Working together to connect food access hubs, such as schools, with the support and resources to provide evidence‐based nutrition education and agricultural literacy can equip individuals and communities with the knowledge, skills, and ability to make nutrient‐rich food choices, helping ensure they live healthfully and thrive.

### CONCLUSIONS

Investments and strategies in nutrition security that utilize the I + PSE model will effectively influence positive behavior change and improve community health. The Let's Eat Healthy initiative is 1 vehicle for activating I + PSE, bringing people together to find creative solutions to address adaptive challenges such as those presented during the COVID‐19 pandemic. Through efforts to improve access to school meals during the onset of the pandemic, innovation to make nutrition education more accessible during extended school closures, and the integration of nutrition education and food access through creative partnerships, Dairy Council of California launched the Let's Eat Healthy initiative to find solutions to improve nutrition and food access in the school environment.

Successful multidisciplinary collaboration that focuses on overcoming adaptive challenges with aligned strategies creates opportunities to positively impact the educational, health, and wellness outcomes for children and families, supporting their nutritional needs within the school environment and beyond. To navigate the challenges in a rapidly changing environment, people and organizations must work together, across disciplines, to leverage knowledge, experience, resources, expertise, and creative thinking. Improving access to healthy food and nutrition education will be most effective when done through collaboration, as organizations can discover and scale innovative solutions to ensure children are supported and have access to the nutritious foods they need.
